# Algal Diet of Small-Bodied Crustacean Zooplankton in a Cyanobacteria-Dominated Eutrophic Lake

**DOI:** 10.1371/journal.pone.0154526

**Published:** 2016-04-28

**Authors:** Ilmar Tõnno, Helen Agasild, Toomas Kõiv, Rene Freiberg, Peeter Nõges, Tiina Nõges

**Affiliations:** Centre for Limnology, Institute of Agricultural and Environmental Sciences, Estonian University of Life Sciences, Tartu County, Estonia; University of Connecticut, UNITED STATES

## Abstract

Small-bodied cladocerans and cyclopoid copepods are becoming increasingly dominant over large crustacean zooplankton in eutrophic waters where they often coexist with cyanobacterial blooms. However, relatively little is known about their algal diet preferences. We studied grazing selectivity of small crustaceans (the cyclopoid copepods *Mesocyclops leuckarti*, *Thermocyclops oithonoides*, *Cyclops kolensis*, and the cladocerans *Daphnia cucullata*, *Chydorus sphaericus*, *Bosmina* spp.) by liquid chromatographic analyses of phytoplankton marker pigments in the shallow, highly eutrophic Lake Võrtsjärv (Estonia) during a seasonal cycle. Copepods (mainly *C*. *kolensis*) preferably consumed cryptophytes (identified by the marker pigment alloxanthin in gut contents) during colder periods, while they preferred small non-filamentous diatoms and green algae (identified mainly by diatoxanthin and lutein, respectively) from May to September. All studied cladoceran species showed highest selectivity towards colonial cyanobacteria (identified by canthaxanthin). For small *C*. *sphaericus*, commonly occuring in the pelagic zone of eutrophic lakes, colonial cyanobacteria can be their major food source, supporting their coexistence with cyanobacterial blooms. Pigments characteristic of filamentous cyanobacteria and diatoms (zeaxanthin and fucoxanthin, respectively), algae dominating in Võrtsjärv, were also found in the grazers’ diet but were generally avoided by the crustaceans commonly dominating the zooplankton assemblage. Together these results suggest that the co-occurring small-bodied cyclopoid and cladoceran species have markedly different algal diets and that the cladocera represent the main trophic link transferring cyanobacterial carbon to the food web in a highly eutrophic lake.

## Introduction

It is well-known that with increasing nutrient loading and fish-predation levels, large-sized crustaceans are replaced by smaller species (e.g. [1, 2]). A similar shift toward smaller crustaceans, especially among cladocerans, occurs with increasing cyanobacterial biomass [3]. This can be explained by the competitive dominance achieved by smaller cladocerans that are less inhibited by toxic cyanobacteria or large filament size [4, 5]. Consequently, crustacean communities dominated by small cladocerans, such as small *Daphnia* species, *Bosmina* and *Chydorus*, and by cyclopoid copepods often prevail in highly eutrophic systems (e.g. [6]). Grazing by such zooplankton (ZP) assemblages, however, is not sufficient to control phytoplankton biomass [1, 7].

Large daphnids that are generalist feeders can affect wide spectra of food types and sizes [8]. Conversely, small cladocerans, and especially cyclopoid copepods, are more specialized feeders, with size and prey-type selection gaining more importance [9, 10]. Kerfoot and Kirk [10] demonstrated strong size selectivity and some taste selectivity in small *Daphnia*, *Diaphanosoma* and *Chydorus*, whereas *Bosmina longirostris* had clearly excercised taste discrimination. While large cladocerans (e.g. large *Daphnia* species) function more as generalist detritivores, recycling both low quality detritus and algae over a certain size range [10], smaller cladocerans may have clear preferences for certain types of living algae. Cyclopoid copepods represent a diverse group of omnivores with species-specific feeding characteristics determining the extents of their carnivory or herbivory. Some cyclopoid species depend largely on animal prey, while others feed and reproduce successfully on pure algal diets [11]. Hopp and Maier [12] demonstrated the morphometric differences of the mouthparts of various freshwater cylopoid species (*Cyclops vicinus*, *C*. *abyssorum*, *Acanthocyclops robustus*, *Mesocyclops leuckarti* and *Thermocyclops crassus*). They concluded that variable distances between setae and setules of maxillipeds of different cyclopoids suggest that these grazers have distinctive algal diet preferences. Rather little is known about small-bodied crustacean consumption of filamentous and colonial cyanobacteria [13]. Although nutritionally inadequate [14], large cyanobacterial stocks in eutrophic water bodies can potentially represent a significant supplementary food source for ZP [15, 16]. A recent field study [17] confirms that phytoplankton assemblages dominated by cyanobacteria (*Microcystis*, *Anabaena*, *Planktothrix*) can be suppressed by grazing of small-sized crustacea, such as cylopoid copepods and small cladocerans. A large amount of freshwater research is currently focusing on the ability of ZP to control phytoplankton growth, and more importantly on control of cyanobacterial blooms and on energy flow pathways from cyanobacteria into food webs (e.g. [18, 19]). However, knowledge is still very scarce on phytoplankton selection and ingestion in nature by small and more selective cladocerans and cyclopoid copepods. As small crustacean species are becoming more important in nutrient- and cyanobacteria-rich systems [3], and are important trophic mediators between primary producers and planktivorous fish, it is important to understand which algal types support the grazing food chain and secondary production in highly eutrophic lakes.

Studying the selective consumption by zooplankton of natural algal assemblages *in situ* is a challenging task. Laboratory experiments can give valuable detailed information on particular grazer and algal species relationships (feeding preferences, grazer resistance, toxicity etc. [11, 20]), but in natural systems ZP feeding and selectivity may depend on the relative availability of different food sources. Mesocosm experiments usually give an assessment of community level grazing impact on algal assemblages, including both the direct removal of cells and indirect effects via nutrient recycling (e.g. [19]). Nevertheless, the actual proportions of algal types ingested by different grazers remains unknown from these experiments. Carotenoid pigment signatures that are phytoplankton-group specific offer a solution. There has been fast progress in applying high performance liquid chromatography (HPLC) of phytoplankton pigments (PPig) for phytoplankton (PP) monitoring (e.g. CHEMTAX) in marine and in freshwater ecosystems [21, 22, 23]. Also, the analysis of gut pigment contents in herbivores has been used widely in marine *in situ* ZP grazing and feeding selectivity studies (e.g. [24, 25, 26]), but much less so in fresh waters (e.g. [27, 28]). The HPLC is an advantageous method for ZP feeding selectivity studies, as it allows determination of the ingestion of small-sized but abundant PP groups for which microscopic identification is problematic [29]. Although the method has also a clear drawback, because so little is known about specific degradation rates of carotenoid pigments in ZP guts, PPig analysis can provide useful information about *in situ* dietary selectivity of freshwater ZP [28], as further elaborated in the Discussion section below.

We applied the HPLC technique to study crustacean gut content for algal marker pigment composition. Our intent was to evaluate qualitatively which PP groups are preferred as food by small-bodied copepod and cladoceran species over a seasonal cycle in eutrophic Võrtsjärv (Estonia), which is dominated by filamentous diatoms and cyanobacteria. We addressed three questions: (1) which PP groups constitute the food of the dominant copepod and cladoceran species over a seasonal cycle; (2) which algae do the small-bodied cladocerans and cylopoids prefer; and (3) what is the significance of cyanobacteria in the diet of small crustaceans and in the trophic transfer to the higher pelagic food chain.

## Materials and Methods

### Study site

Lake Võrtsjärv is a large (270 km^2^), shallow (mean depth 2.8 m; maximum depth 6 m) and eutrophic lake in southern Estonia. Mean concentrations of total N and P are 1.6 mg l^-1^ and 0.053 mg l^-1^, respectively. The ice cover lasts on average 135 days, from November to April. Because it is shallow, the lake is constantly mixed to the bottom, keeping the water turbid throughout the ice-free period [30]. Filamentous cyanobacteria and diatoms dominate by biomass in the PP community, accompanied by smaller algae such as chlorophytes, cryptophytes and chrysophytes [31]. Cladocerans and cyclopoid copepods are the dominant ZP groups by biomass, while rotifers dominate in Võrtsjärv by numbers [32].

### Sampling

Data for the present study were gathered from February 2010 to February 2011 from a regular monitoring point in the deepest part of Võrtsjärv (maximum depth 6 m). Water samples were collected fortnightly (in May and from July to October) or monthly (during rest of the year). For depth-integrated samples, water collected at 1-m intervals with a Ruttner sampler from the entire water column (3–4 m depending on the water level) was mixed in a tank. From this depth-integrated water a subsample was taken to analyse phytoplankton composition and biomass. For ZP composition and biomass analyses, 10 L of the depth-integrated water were filtered through a 48 μm plankton net. Phytoplankton and ZP samples were fixed with acidified Lugol’s solution at a final concentration of 1% and kept in the dark. Additional depth-integrated samples for analysing PPig in metazooplankton guts were collected with vertical tows of the plankton net (48 μm) until sufficient amounts of material were gathered. The collected ZP was immediately filtered gently through a 300 μm plankton net and rinsed with filtered lake water to remove as much phytoplankton as possible. The cleaned zooplankton was anaesthetized in carbonated water and concentrated in a small volume of filtered lake water. Samples for analysis of PPig in ZP guts were stored frozen (-20 C°) and in darkness prior to analyses. No specific permissions were required for any part of the study, and field studies did not involve endangered or protected species.

### Plankton biomass and ZP sorting

Utermöhl’s technique [33] was used to analyse PP biomass. Cells were enumerated with an inverted microscope (Ceti Versus, Belgium) at ×400 magnification. Phytoplankton taxa were identified to the species level whenever possible. Counts of each taxon were converted to biovolumes by measuring cell/trichome/colony dimensions and approximating each taxon with a simple geometric shape. Biovolume was converted to biomass (wet weight, g WW m^-3^) assuming the specific weight of algal cells is approximately equal to that of water [34]. Phytoplankton cells (<30 μm) presumably ingestible for ZP in respect to size [7] were distinguished in the counting results (S1 Table).

The ZP were counted in triplicate subsamples (2.5 or 5 ml) under a dissecting microscope (Olympus SZ40, Germany) at ×60 magnification. The weights of individual rotifers were estimated from average lengths according to Ruttner-Kolisko [35]. The lengths of crustaceans were converted to wet weights according to Studenikina and Cherepakhina [36] for nauplii, and to Balushkina and Winberg [37] for other groups (S2 Table).

Before PPig analyses, frozen ZP samples were thawed and, the most abundant ZP species were separated. Among copepods we sorted three cyclopoids (*Cyclops kolensis*, *Mesocyclops leuckarti* and *Thermocyclops oithonoides*) and among cladocerans three dominant taxa (*Daphnia cucullata*, *Chydorus sphaericus* and *Bosmina* spp.). The zooplankton specimens were individually picked by using forceps under the dissecting microscope and their bodies checked for externally attached PP cells or filaments. Only clean indiviuals were included in analyses. From each subsample, 100–300 individuals of each species were separated, rinsed once more to minimize contamination from non-ingested algae and collected on Whatman GF/F glass microfibre filters (0.7 μm) for pigment extraction. For copepod analyses, mainly adult females and later stages of copepodites were used. Whenever possible, replicate samples were analysed (S3 Table).

### Pigment extraction and HPLC analyses

Considering the dominance of cyanobacteria and diatoms in Võrtsjärv PP, marker pigments of cyanobacteria such as zeaxanthin (Zea; mainly from filamentous forms) and canthaxanthin (Cantha; from colonial forms), and marker pigments of diatoms—fucoxanthin (Fuco), diadinoxanthin (Diadino), and diatoxanthin (Diato)—were analysed [31, 38, 39]. Both Diato and Diadino were selected because Diadino can be transformed to Diato in the xanthophyll cycle of diatoms in strong light [40]. Lutein (Lut) and chlorophyll *b* (Chl *b*) were analysed as proxies of chlorophytes, while the carotenoid alloxanthin (Allo) represented cryptophytes. Chlorophyll *a* (Chl *a*) was chosen as a proxy of total PP biomass [41]. For analysed pigments the abbreviations above were recommended by Roy et al. [41].

The analyses of PPig followed the protocols of Leavitt and Hodgson [42], Quiblier-Lloberas et al. [27], and Lie and Wong [43]. Depth integrated water samples (60–200 ml, depending on water turbidity) and sorted ZP suspensions were filtered through Whatman GF/F filters and frozen (-80°C) until PPig analysis. Solution of 90% acetone (by volume) was added to frozen GF/F glass fibre filters to extract PPig, thereafter samples were sonicated (Branson 1210) for about 10 min in an ice-bath under dim light. All samples were extracted at -20°C in the dark for 24 h. To remove any particles, the pigment extracts were clarified by filtration through a 0.45 μm filter (Millex LCR, Millipore).

To separate PPig, reversed-phase high performance liquid chromatography (RP-HPLC) was applied, using a Shimadzu Prominence (Japan) series binary gradient system with a photodiode array (PDA) and fluorescence detector (see Tamm et al. [23] for more details). For peak identification and quantification, we used commercially available external standards from DHI (Denmark).

### Statistical analyses and selectivity calculations

To test relationships between water column pigments and biomass of different PP groups, we used Pearson correlation coefficients. To examine differences between pigment proportions in phyto- and zooplankton, a simple t-test was performed.

We investigated feeding selectivity of ZP by calculating the alpha selectivity index of Chesson [44], the formula for which is:

$${ɑ}_{i}=\frac{r_{i}/p_{i}}{\sum_{i=1}^{n}\;r_{i}/p_{i}};\;\;i=1\ldots n$$

where r_i_ is the percentage of i-th PPig in ZP guts, p_i_ the percentage of the same PPig in the environment and n the total number of pigments analysed. Chesson’s selectivity index was calculated for eight PPig (except Chl *a*). When ɑ = 1/n (in our study 1/n = 0.125), feeding is non-selective. Values of ɑ_i_ > 0.125 or ɑ_i_ < 0.125 were taken, respectively, to represent selection and avoidance of pigments by ZP.

All statistical analyses (except Chesson’s selectivity index) were performed using STATISTICA 10 [45].

## Results

### Plankton seasonal dynamics

**Fig 1:**
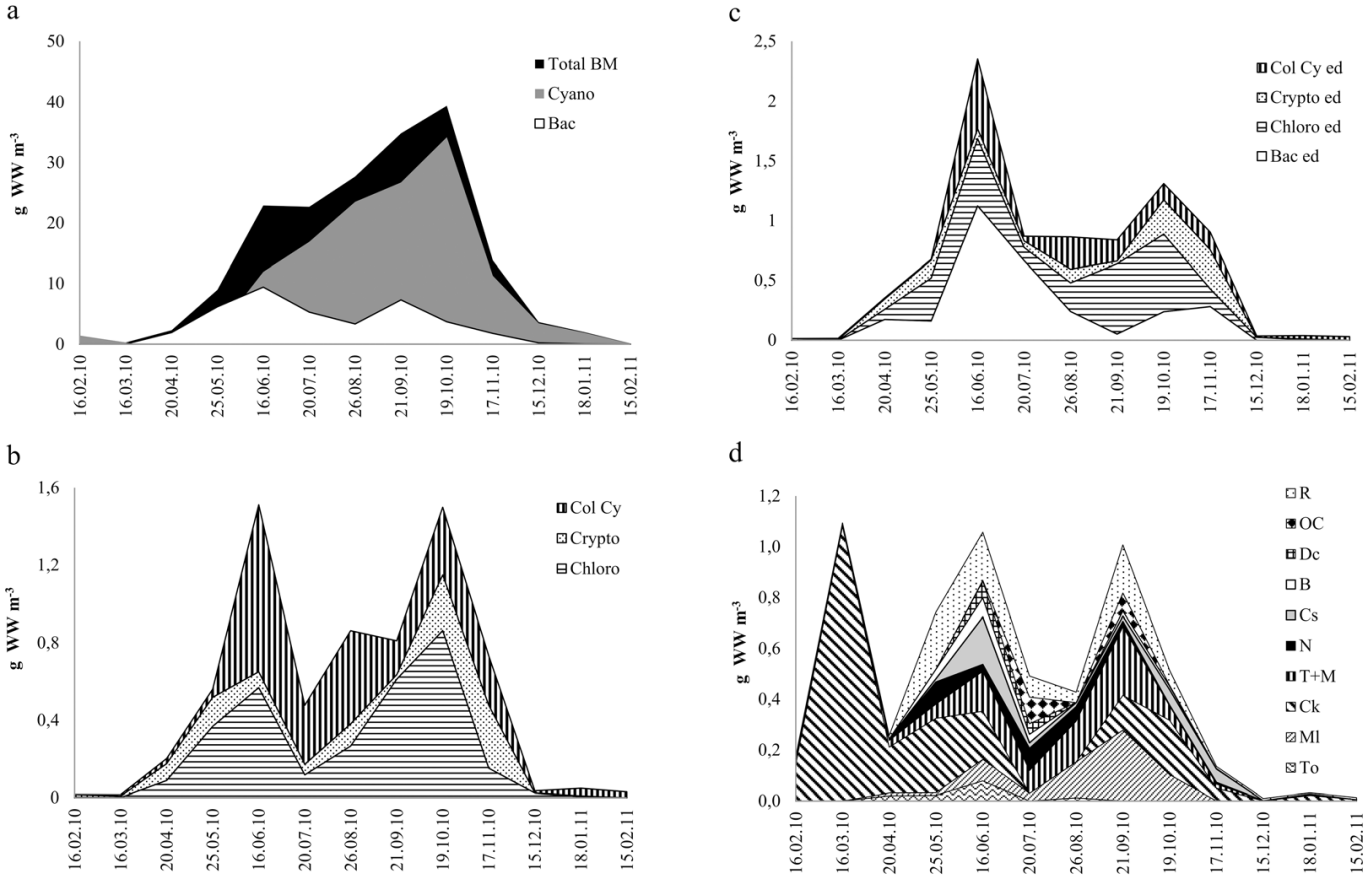
Seasonal dynamics of different phytoplankton and zooplankton groups (g WW m^-3^) in Lake Võrtsjärv during February 2010 to February 2011. (a) Total BM—total phytoplankton biomass; Cyano—cyanobacteria; Bac—diatoms. (b) Chloro—chlorophytes (green algae); Crypto—cryptophytes; Col Cy—colonial cyanobacteria. (c) seasonal dynamics of phytoplankton edible for zooplankton (ed—edible). (d) To–*Thermocyclops oithonoides;*Ml–*Mesocyclops leuckarti*; Ck–*Cyclops kolensis*; T+M—Thermoc.+ Mesoc. copepodites; Cs–*Chydorus sphaericus*; B–*Bosmina* spp.; Dc–*Daphnia cucullata*; OC—Other Cladocerans; N—Nauplii; R—Rotifers. WW—wet weight.

Total PP biomass started to increase in April after the ice break-up and reached its maximum in October, decreasing rapidly thereafter (Fig 1a). Phytoplankton was dominated by the filamentous cyanobacteria *Limnothrix planktonica* and *L*. *redekei* forming up to 90% of PP biomass. Diatoms, the second largest group composed mainly of *Aulacoseira* spp. and *Synedra* spp., had two peaks—in June and September. The mean length of dominating filamentous cyanobacteria and diatoms in Võrtsjärv varied substantially with season, between 60–200 μm and 90–400 μm, respectively. The cyanobacteria filaments were significantly longer in winter than in summer (p< 0.001). Among minor algal groups (Fig 1b), the biomass of chlorophytes (mainly *Pediastrum* spp. and *Scenedesmus* spp.) had two peaks, in May and October, while higher biomass levels of cryptophytes (represented by *Cryptomonas* spp. and *Rhodomonas* sp.) occured in October and November. The biomass of colonial cyanobacteria (mainly *Microcystis*, *Chroococcus*, and *Cyanodictyon*) increased in June and stayed more or less at the same level until November, thereafter decreasing rapidly.

The small PP (<30 μm) constituted on average 13% (from 3% to 63%) of the total phytoplankton biomass and its amount peaked in June and October (Fig 1c). This fraction consisted mainly of diatoms and green algae, with cryptophytes dominating in winter, diatoms and colonial cyanobacteria in summer and green algae in autumn.

**Fig 2:**
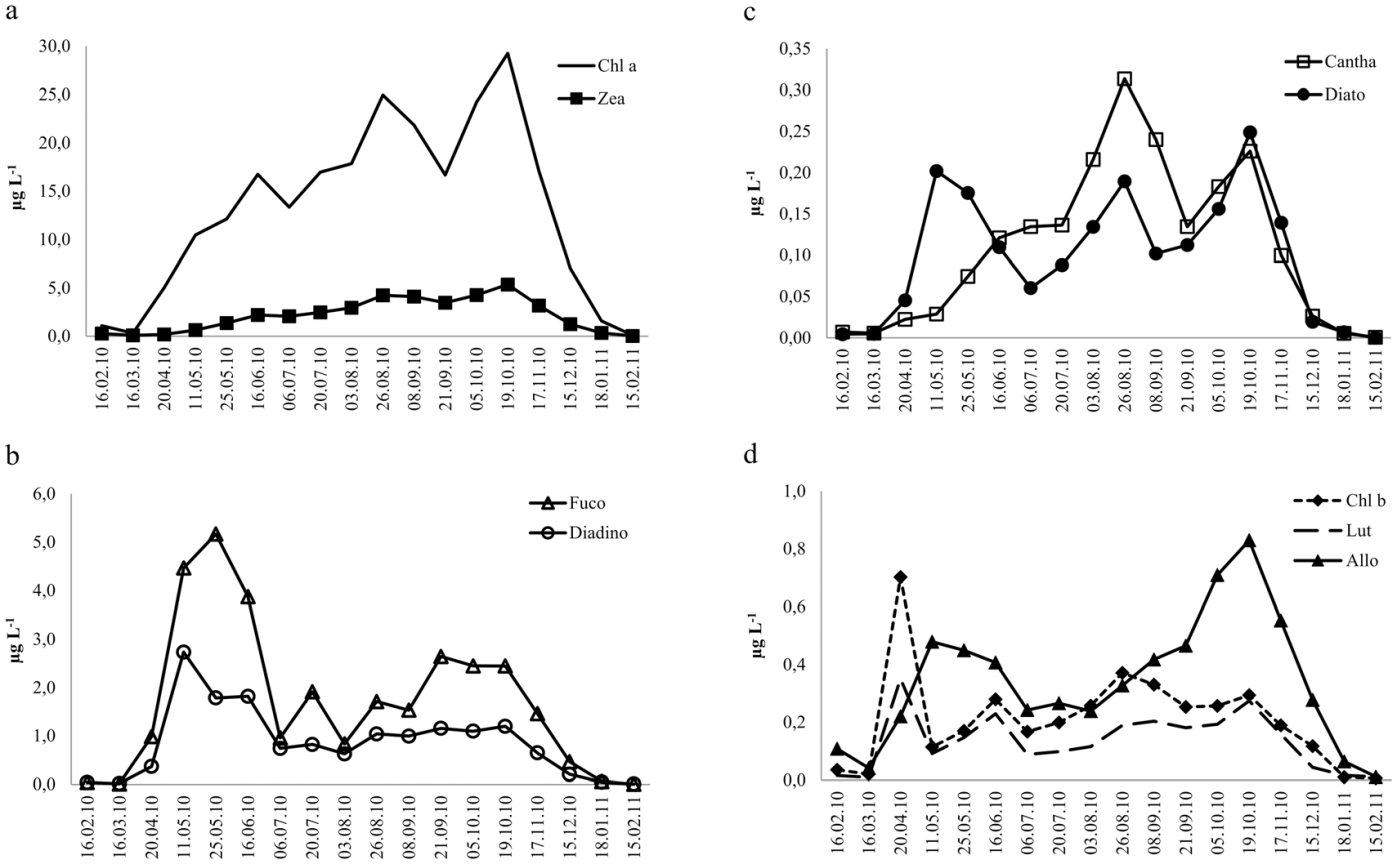
Seasonal dynamics of phytoplankton pigments (μg l^-1^) in Lake Võrtsjärv water column during February 2010 to February 2011. (a) Chl *a–*chlorophyll *a*; Zea—zeaxanthin. (b) Fuco—fucoxanthin; Diadino—diadinoxanthin. (c) Cantha—canthaxanthin; Diato—diatoxanthin. (d) Chl *b–*chlorophyll *b*; Lut—lutein; Allo—alloxanthin.

The dynamics of the studied PPig in lake water generally followed the seasonal succession of PP groups in Võrtsjärv (Table 1; Fig 2a–2d).

**Table 1:** Correlations between investigated water column pigments and phytoplankton groups in Lake Võrtsjärv (02.2010–02.2011).

Pair of characteristics				
Chl *a* & Tot BM	y = -1.35+ 1.35* x	*r* = 0.93	p = 0.00001	*r*^*2*^ = 0.86
Fuco & Diatoms	y = 0.36+ 1.51* x	*r* = 0.87	p = 0.0003	*r*^*2*^ = 0.75
Diadino & Diatoms	y = -0.13+ 4.48* x	*r* = 0.92	p = 0.00001	*r*^*2*^ = 0.85
Diato & Diatoms	y = 1.04 + 22.50* x	*r* = 0.58	p = 0.0365	*r*^*2*^ = 0.34
Zea & Cyano	y = -1.51+ 6.29* x	*r* = 0.95	p = 0.00000	*r*^*2*^ = 0.91
Cantha & Col Cyano	y = -0.013+ 1.56* x	*r* = 0.99	p = 0.00000	*r*^*2*^ = 0.97
Lut & Chloro	y = 0.032+ 1.57* x	*r* = 0.63	p = 0.0220	*r*^*2*^ = 0.39
Chl *b* & Chloro	y = 0.14 + 0.47* x	*r* = 0.32	p = 0.28	*r*^*2*^ = 0.11
Allo & Crypto	y = -0.028+ 0.38* x	*r* = 0.82	p = 0.0007	*r*^*2*^ = 0.67

Chl *a–*chlorophyll *a*; Fuco—fucoxanthin; Diadino—diadinoxanthin; Diato—diatoxanthin; Zea—zeaxanthin; Cantha—canthaxanthin; Lut—lutein; Chl *b–*chlorophyll *b*; Allo—alloxanthin; Tot BM—total phytoplankton biomass; Cyano—cyanobacteria; Col Cyano—colonial cyanobacteria; Chloro—chlorophytes (green algae); Crypto—cryptophytes.

Although the crustacean biomass usually peaks during the warm season (June; [32]), we recorded high abundance (30 ind L^-1^) of crustaceans and the yearly maximum metazooplankton biomass already in March (Fig 1d), with *Cyclops kolensis* being the only crustacean in the plankton until the ice break-up in April. From April onward, thermophilic *M*. *leuckarti* and *T*. *oithonoides* appeared in the plankton, and *M*. *leuckarti* dominated among copepods until the end of the year. Starting from May, cladocerans (mainly *C*. *sphaericus*, *D*. *cucullata* and *Bosmina* spp.) were established in the plankton. Their highest biomass occurred in June dominated by *C*. *sphaericus*. Among rotifers, *Keratella* spp., *Polyarthra* spp. and *Anuraeopsis fissa* were the dominant taxa forming highest densities in June and July.

### Grazing selectivity and feeding seasonality of zooplankton

**Fig 3:**
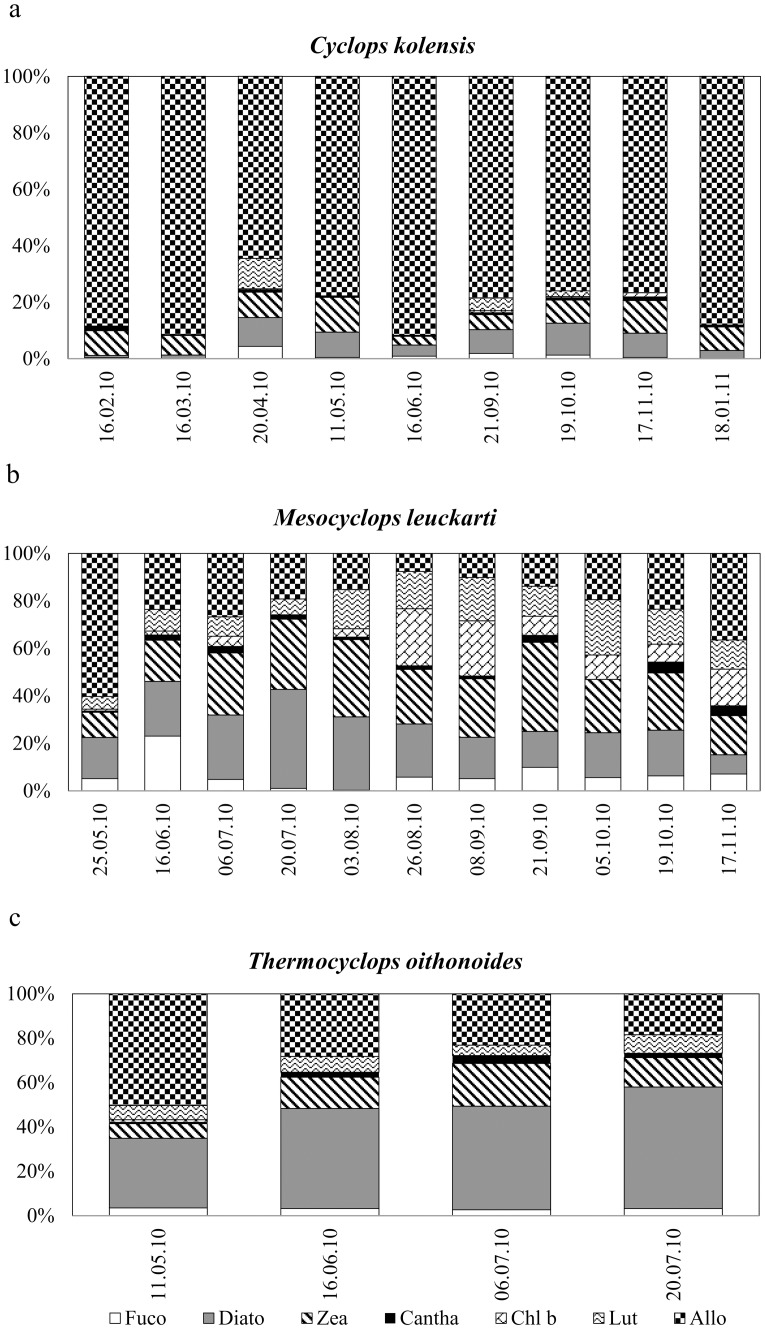
Percentage contribution of phytoplankton pigments in cyclopoid copepods’ guts. (a) *Cyclops kolensis*. (b) *Mesocyclops leuckarti*. (c) *Thermocyclops oithonoides*. Abbreviations of phytoplankton pigments as in Fig 2.

Crustacean species were analysed for pigment compositions according to their seasonal appearance in the plankton. Throughout the presence of *C*. *kolensis* in the plankton, its gut contained predominantly the cryptophyte marker pigment Allo, whereas the pigment compositions in the other copepods, mainly occurring in summer, were more variable (Fig 3a–3c). The proportions of Allo and Diato were higher and the proportions of Fuco, Diadino and Chl *b* were lower in the guts of *C*. *kolensis* compared to the lake PP assemblage (*t*- test, p< 0.05), indicating selective feeding by *C*. *kolensis*. The differences of the proportions of other investigated pigments between *C*. *kolensis* guts and lake water were statistically non-significant (Table 2). Chesson’s selectivity index demonstrated clear preference of Allo, but also Diato in the *C*. *kolensis* diet during the study period (Table 3). The feeding of *C*. *kolensis* was most variable in April when Fuco, Diato, Zea and Lute were also present in its guts (Fig 3a). In the guts of *M*. *leuckarti*, the proportions of Diato, Chl *b*, Lute and Allo were higher and the proportions of Fuco and Diadino lower than in the lake PP assemblage. Variable pigment composition in *M*. *leuckarti* guts was detected in July, August, September 21, and October 5 (Table 2; Fig 3b). The guts of *T*. *oithonoides* contained mainly the marker pigments Allo and Diato, with less Lute and Zea. In May, the diet of *T*. *oithonoides* contained mainly Allo, but it was more variable in June and July (Fig 3c). According to the selectivity index, copepods *M*. *leuckarti* and *T*. *oithonoides* preferred cryptophytes, diatoms and chlorophytes in their diets (Table 3).

**Table 2:** Results of *t*-tests between proportions of the investigated phytoplankton pigments in phytoplanton (PP) and most abundant cyclopoid species (*Cyclops kolensis* and *Mesocyclops leuckarti*) in Lake Võrtsjärv (02.2010–02.2011).

	*t*	p
*Cyclops kolensis*		
Fuco	-3.94	0.0007
Diadino	-4.49	0.0002
Diato	4.41	0.0002
Zea	-1.22	NS
Canta	1.63	NS
Chl *b*	-2.97	0.007
Lute	0.52	NS
Allo	20.74	0.0000
*Mesocyclops leuckarti*		
Fuco	-2.66	0.0131
Diadino	-7.88	0.0000
Diato	7.86	0.0000
Zea	2.04	NS
Canta	1.67	NS
Chl *b*	3.18	0.0037
Lute	8.00	0.0000
Allo	4.00	0.0005

*t*< 0: pigment proportion is lower in zooplankton (ZP) than in PP; *t*> 0: pigment proportion is higher in ZP than in PP. NS—not significant. Abbreviation of phytoplankton pigments as in Table 1.

**Table 3:** Chesson’s selectivity index 〈 of investigated zooplankton (ZP) species for phytoplankton pigments within the period from 02. 2010 to 02. 2011 in Lake Võrtsjärv.

Date	Fuco	Diadino	Diato	Zea	Cantha	Chl *b*	Lute	Allo
*Cyclops kolensis*								
16.02.10	0.007	0	**0.138**	0.026	0.177	0	0	**0.651**
16.03.10	0	0	0.098	0.033	0.022	0	0	**0.848**
20.04.10	0.007	0	**0.351**	0.079	0.056	0.001	0.048	**0.458**
11.05.10	0	0	**0.182**	0.079	0.073	0	0	**0.665**
16.06.10	0.001	0	**0.136**	0.005	0.011	0	0	**0.847**
21.09.10	0.002	0	**0.270**	0.005	0.018	0.010	0.090	**0.605**
19.10.10	0.003	0	**0.299**	0.010	0.023	0.011	0.048	**0.605**
17.11.10	0.001	0	**0.273**	0.016	0.058	0.034	0	**0.617**
18.01.11	0	0	**0.219**	0.013	0.062	0	0	**0.707**
*Mesocyclops leuckarti*								
25.05.10	0.003	0	**0.340**	0.027	0.026	0.016	**0.126**	**0.463**
16.06.10	0.017	0	**0.607**	0.023	0.053	0.017	0.115	**0.168**
06.07.10	0.007	0	**0.628**	0.018	0.029	0.036	**0.129**	**0.154**
20.07.10	0.001	0	**0.740**	0.019	0.020	0.001	0.107	0.113
03.08.10	0.001	0	**0.494**	0.024	0.010	0.030	**0.306**	**0.137**
26.08.10	0.011	0	**0.391**	0.018	0.016	**0.241**	**0.274**	0.076
08.09.10	0.009	0	**0.462**	0.016	0.013	**0.191**	**0.243**	0.066
21.09.10	0.012	0	**0.444**	0.036	0.072	0.106	**0.231**	0.099
05.10.10	0.007	0	**0.382**	0.017	0	**0.126**	**0.381**	0.086
19.10.10	0.012	0	**0.364**	0.021	0.095	0.122	**0.253**	**0.133**
17.11.10	0.015	0	**0.173**	0.016	0.122	**0.244**	**0.232**	**0.198**
*Thermocyclops oithonoides*								
11.05.10	0.002	0	**0.415**	0.028	0.060	0.027	**0.187**	**0.281**
16.06.10	0.002	0	**0.765**	0.012	0.035	0	0.058	**0.129**
06.07.10	0.003	0	**0.805**	0.010	0.027	0	0.056	0.099
20.07.10	0.002	0	**0.780**	0.007	0.018	0	0.106	0.087
*Daphnia cucullata*								
16.06.10	0.019	0.046	0.117	0.035	**0.443**	0	**0.281**	0.059
06.07.10	0.061	0.117	**0.142**	0.067	**0.567**	0.008	0	0.037
20.07.10	0.018	0.097	0.124	0.038	**0.528**	0	**0.135**	0.060
*Bosmina* spp.								
16.06.10	0.061	0	**0.164**	0.032	**0.415**	0	**0.244**	0.085
06.07.10	0.024	0	**0.508**	0.027	**0.218**	0	**0.153**	0.071
*Chydorus sphaericus*								
20.07.10	0.004	0.021	0.103	0.008	**0.773**	0.013	0.048	0.030
03.08.10	0	0	0.085	0.009	**0.740**	0.015	0.043	0.107
17.11.10	0	0	0	0.005	**0.967**	0.008	0	0.021

Values indicating positive selection (ɑ_i_ > 0.125) are marked in bold. 0–phytoplankton pigment was not detected in ZP guts. Abbreviations of phytoplankton pigments as in Table 1.

**Fig 4:**
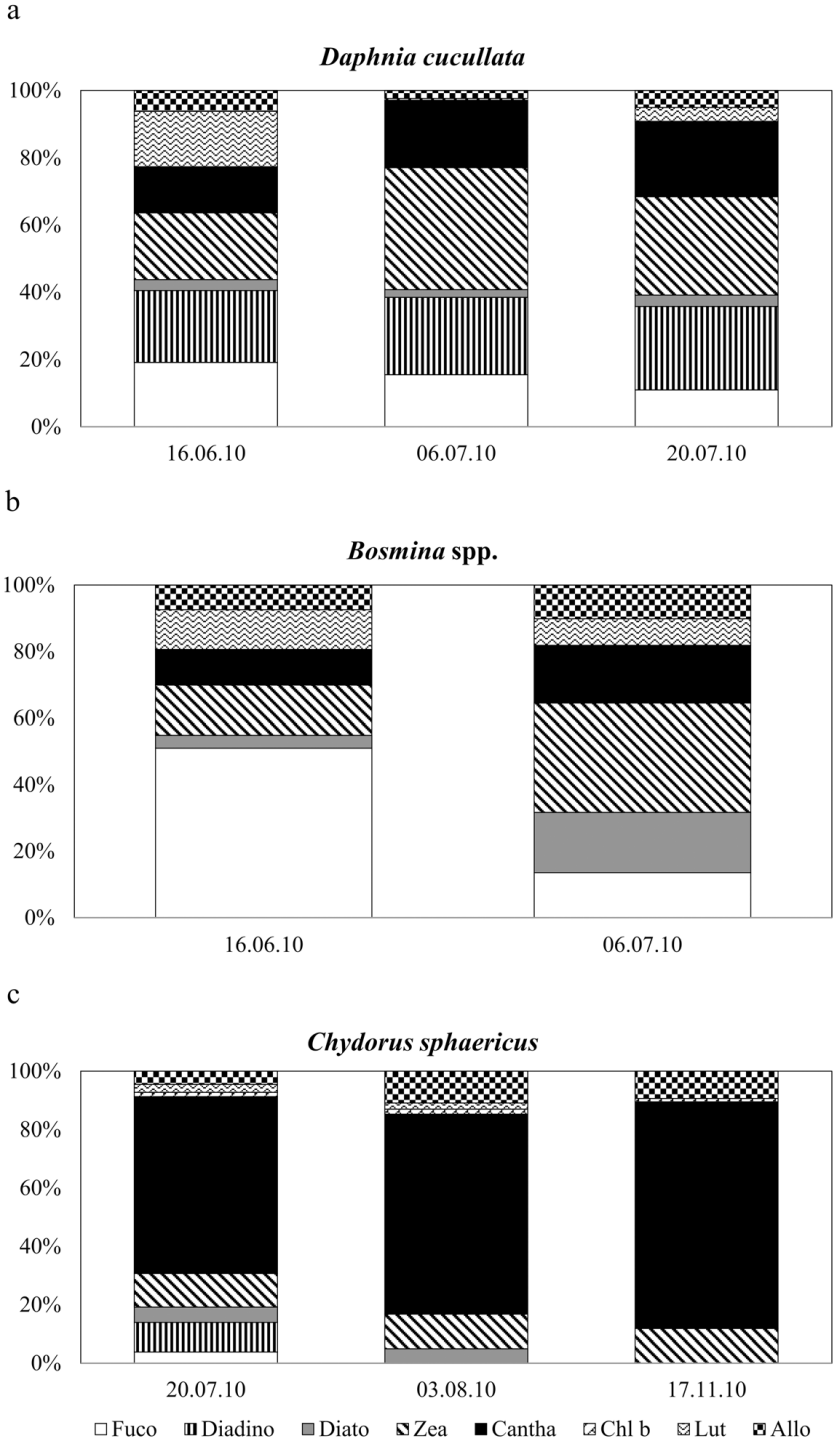
Percentage contribution of phytoplankton pigments in cladoceran guts. (a) *Daphnia cucullata*. (b) *Bosmina* spp. (c) *Chydorus sphaericus*. Abbreviations of phytoplankton pigments as in Fig 2.

The cladoceran samples covered mainly the summer period. The guts of *D*. *cucullata* and *Bosmina* spp. contained mostly marker pigments of diatoms and cyanobacteria (Fig 4a and 4b), while those of *C*. *sphaericus* contained overwhelmingly the colonial cyanobacterial carotenoid Cantha (Fig 4c). All investigated cladocerans had the highest positive feeding selectivity for colonial cyanobacteria, but it was high also for diatoms (Diato) and clorophytes (Table 3).

Chesson’s selectivity indices revealed that all ZP species studied avoided filamentous diatoms (Fuco) and cyanobacteria (Zea) in their diets, and the copepods also avoided colonial cyanobacteria (Table 3).

## Discussion

We are well aware that our study has the limitation that we did not conduct extra measurements for pigment-specific degradation rates in ZP guts. We were unable to find consistent rates from the literature, because the results tend to be controversial: some studies show pigments fully preserved in copepods, cladocerans and other small zooplankton guts [27, 46], while other show remarkable degradation of carotenoids [47, 48]. Like Van Nieuwerbrugh et al. [49], we assumed that Chesson’s selectivity index can be considered a snapshot based on pigments of PP transferred through ZP to the food web, and that this transfer is presumably much faster than digestive changes in the composition of carotenoid pigments. Also, it is important to note that this study focuses on dietary selectivity by ZP (a qualitative study) and not on ZP grazing rate (a quantitative study). The pigment degradation information would be crucial for a grazing-rate study, as discussed by Thys et al. and Pandolfini et al. [28, 50].

### Phytoplankton preferences of copepods and cladocerans

Major questions underlying the research were the levels of selectivity by smaller planktonic crustacea for cyanobacteria and the contribution of this dominant algal source to ZP diets in a highly eutrophic lake. Cyanobacteria in Võrtsjärv are predominantly filamentous and exceed the presumably edible size range for ZP [7]. Regarding copepods, our results confirmed small contributions of filamentous cyanobacteria but only a negligible contribution of colonial cyanobacteria to the diets of the summer cyclopoids *M*. *leuckarti* and *T*. *oithonoides*. Based on the selectivity index, all of the investigated copepods (*C*. *kolensis*, *M*. *leuckarti* and *T*. *oithonoides*) avoided ingesting cyanobacteria. *Mesocyclops leuckarti* had the most diverse algal diet and, unlike the other two cyclopoids, its guts contained a relatively high percentage of Zea. Chlorophytes contain also Zea as a minor pigment, some of that detected in cyclopoid guts could have originated from more easily consumed green algae. One should keep in mind that some carotenoids such as Zea and Cantha could be also body pigments of copepods [42]. However, relative to Zea and Cantha, Astaxanthin is generally the main body pigment of copepods [49].

To date the limited information on cyclopoid feeding on cyanobacteria has been controversial. In experiments with filamentous cyanobacteria (*Cylindrospermopsis raciborskii*, *Anabaena solitaria* and *A*. *flos-aquae*) *Mesocyclops ogunnus* did not ingest any of the offered species [13]. In a study by Work and Havens [15], both filamentous and colonial cyanobacteria were found in the guts of cyclopoids, although they were less common than in calanoid copepods and in *Daphnia* spp. Recent community-level investigations in eutrophic Lake Ringsjön (Sweden) have shown that a cyclopoid-dominated community can significantly supress the growth of *Anabaena*, *Microcystis* and *Planktothrix* species during the period of a cynaobacterial bloom [17].

Given the general avoidance of cyanobacteria in present study, the cyclopoids probably are not able to negatively affect the filamentous or colonial cyanobacteria in Võrtsjärv. Instead, diatoms generally were the algae most preferred by *M*. *leuckarti* and especially by *T*. *oithonoides* during the cyanobacterial dominance. The second algal group preferred by all cyclopoids were the cryptophytes, which were an especially important food for *C*. *kolensis* that appears mainly in winter. According to the high Allo contributions (64–92% of the ingested pigments), the guts of *C*. *kolensis* were packed with cryptophytes. Cryptophytes contain essential fatty acids and represent high-quality zooplankton food, enhancing growth and productivity of herbivorous ZP even in low amounts [51]. Thus, the diet of *C*. *kolensis* suggests the selection of the more nutritious algae from among the phytoplankton assemblage [14]. Similarly, several other studies have also concluded that phytoflagellates like cryptophytes are ingestible and preferred algae for rotifers [52], cladocerans [28] and ciliates [53]. Moreover, cryptophytes have been suggested to form an essential food base for survival and development of copepod nauplii [54].

We observed clear dietary differences between winter (*C*. *kolensis*) and summer copepod species (*M*. *leuckarti* and *T*. *oithonoides*). The more variable algal diets of thermophilic *M*. *leuckarti* and *T*. *oithonoides* may be partly determined by the phytoplankton succession during their growing period. Still, the dietary difference between these two cyclopoid species, which occur in the plankton together in June, suggests there is species-specific food selection [12].

Having different feeding modes, specifically filter-feeding, the cladocerans selected other algal groups than did the raptorially feeding cyclopoid copepods [11, 55]. Although Allo was present in the guts of the cladocerans, no selectivity for cryptophytes occurred; instead more copious gut contents and selectivity for colonial cyanobacteria, unlike copepods, were evident in all the taxa studied (*D*. *cucullata*, *Bosmina* spp., *C*. *sphaericus*) and on all dates analysed. *Bosmina* and *Daphnia* also showed high selectivity for chlorophytes and diatoms, which was not the case for *Chydorus*. Although filamentous cyanobacteria in Võrtsjärv probably interfere with cladoceran feeding (i.e. a majority of the filaments are inedible, with mean filament lengths of 60–200 μm) [56], they may still occasionally be ingested when appearing among the filtered food, especially if the filaments are small [13]. It is difficult to assess by our present methodology whether live filaments or detritus originating from filamentous cyanobacteria contributed most of the Zea in cladoceran gut contents. Filamentous cyanobacteria (mostly *Limnothrix* spp., *Planktolyngbya limnetica* and *Aphanizomenon skujae*) dominate in Võrtsjärv during summer, and they probably contribute most to detritus formation [31]. Due to their abundance, detrital particles can represent an important food resource for less discriminating suspension feeders such as daphnids [10]. Still, considering the proportions of Zea and Cantha in the cladocerans’ guts, approximately half of the algal diet of *D*. *cucullata* and *Bosmina* spp. might originate from different forms of cynaobacteria.

The smallest analysed cladoceran, *Chydrous sphaericus*, had a strong positive selection only for colonial cyanobacteria, suggesting feeding behaviour different from that of compared to the other tested cladocerans. *Chydorus sphaericus* is characteristic of eutrophic water bodies, and it is better adapted than other small-bodied cladocerans to eutrophic conditions with abundant detritus and high cyanobacterial concentrations [57]. In Võrtsjärv it is the most abundant cladoceran species, contributing up to 38% of the total zooplankton biomass during its growing period (H. Agasild unpubl.). Although the number of samples analysed for *C*. *sphaericus* was limited in our study, the high contribution to its gut pigment content from colonial cyanobacteria (more than 70% of the ingested pigments) showed that this algal food was essential for this cladoceran throughout different seasons. Unlike other Chydoridae, that live in the littoral, *C*. *sphaericus* is adapted to life in the pelagic zone, where it frequently attaches to detrital particles and fascicles of algae [58]. Attached to algal colonies, it feeds by scraping the colony surface using its thoracic limbs, collecting the small and easily detached cells in its filtering chamber. This behaviour may explain the high content of Cantha in the diet of *Chydorus*. Still, colonial cyanobacteria represent a minority phytoplankton group in Võrtsjärv [31]. Among colonial cyanobacteria, the dominant colonies of *Microcyctis pulverea* and *Cyanodicton* spp. are composed of ca 2 μm cells that are easily ingestible for *Chydorus*, as has been concluded from zooplankton feeding experiments with fluorescent microparticles [59]. Although cyanobacteria are considered to provide lower-value food for ZP reproduction compared to green algae or cryptophytes [14], previous studies in Võrtsjärv have shown that *C*. *sphaericus* also feeds efficiently on bacteria and micro-ciliates [59, 60], and it probably could utilize these sources as a significant food supplement. Owing to its high abundance, *C*. *sphaericus* is the major grazer on small algal cells, bacteria and ciliates in Võrtsjärv, relative to the other cladocerans studied [59, 60]. The behaviour of *Chydorus* and its gut content observed in the present study support the conclusion that colonial cyanobacteria provide abundant and easily accessible food for *Chydorus* in the pelagic zone of lakes, which may also explain its success and dominance in waters with high concentrations of cyanobacteria.

According to our results, some of the small cladocerans dominant in highly eutrophic waters are not avoiding ingestion of colonial cyanobacteria, which can serve as one significant energy source in the grazing food chain. However, the preferential feeding of *C*. *sphaericus* on colonial cyanobacteria observed in this study still requires confirmation by further studies in order to determine the scale of its role in controlling the stocks of cyanobacteria.

### Algal pathways and seasonal importance of algal sources to the grazing food chain

Previous zooplankton grazing studies in Võrtsjärv have revealed a relatively low metazooplankton feeding impact on total PP biomass [7, 61]. The daily consumption was on average only 4% of the total PP biomass. Stronger grazing pressure was estimated for a small-sized phytoplankton fraction (<30 μm), proportion of which in the total PP biomass were, consequently, kept low [7]. Presumably these smaller cells represent the most important algal food source for grazing zooplankton. Among the phytoplankton assemblage, smaller cells or colonies (mostly among <30 μm [56, 62]) are generally considered more palatable and preferred food for zooplankton compared to algal aggregates or long chains of cells. The present study gives further insight into algal preferences of crustaceans, and some general conclusions can be drawn regarding seasonal shifts in phytoplankton support of the grazing food chain in a temperate, eutrophic lake.

Two distinct seasonal periods can be described in a temperate lakes: cold and ice-covered and warmer open-water periods. In the colder period with limited, small-sized, ‘edible’ phytoplankton biomass, cryptophytes were the dominant zooplankton food, contributing most to the crustacean diet from November to May. During that period the crustacean community was mostly copepods. Beside availability, additional characteristics of cryptophytes, such as motility of the cells, probably attract copepods. The motility is especially important for detection of prey by raptorial feeders such as cylopoid copepods [9]. Cyclopoids can also be largely carnivorous, preying on herbivores, such as rotifers, small cladocerans, copepod nauplii and ciliates [54, 56, 63]. Thus, the copepods’ gut-pigment composition may at least partly originate from their prey organisms. Throughout our study, all cryptophytes (mostly *Cryptomonas* sp. and *Rhodomonas* sp. with cell diameters of 12–36 μm and 6 μm, respectively) belonged to the easily ingestible size range for zooplankton and likely provided food for all herbivorous grazers. Given the substantial contribution of Allo to the dietary pigments in the dominant cyclopoid species in Võrtsjärv (whether via direct algal consumption or indirectly via prey), cryptophytes appear to be an important algal source for the planktonic grazing food chain in this lake during the colder season.

Beginning in June, diatoms and especially cyanobacteria formed the bulk of phytoplankton biomass in Võrtsjärv, while the small-sized (< 30 μm) ‘edible’ fraction was dominated mostly by diatoms, green algae and colonial cyanobacteria. Along with changed algal and grazer composition during the warm period, a greater variety of phytoplankton groups, including cryptophytes, cholorophytes and diatoms together with cyanobacteria, was observed to contribute to the algal diet of crustaceans. This was particularily evident when filter-feeding cladocerans appeared in the plankton, grazers with clearly different feeding preferences (major diet contributions from cyanobacteria) compared to raptorial cyclopoids (with more diverse algal diets). Thus, the different feeding modes of cladocerans and cyclopoids seem to provide diverse algal diet niches. Considering the marked observed differences in algal pigment compositions among the dominant, small-sized copepod and cladoceran species found in present study, the seasonal fluctuations in the crustacean community presumably influence the relative proportions of algal sources channelled into the food web. According to our results, cladocerans seem to be the major mediators transferring cyanobacterial production to higher trophic levels in Võrtsjärv. Thus, in periods with higher cladoceran biomass and *Chydorus* dominance in the community, such as early summer and autumn [32], cyanobacteria could represent a significant carbon source to the grazing food chain in this large, shallow lake.

In conclusion, although small-bodied crustaceans are typical for eutrophic waterbodies generally, they are unable to control phytoplankton growth, and they have clear selectivities for particular algae in the phytoplankton assemblages. Our study showed considerable variations in the algal diet composition and preferences among the dominating cyclopoid and cladoceran species co-occurring in the plankton. The results also confirmed that colonial cyanobacteria could represent an important food source to small-bodied cladoceran community, especially when dominated by *Chydorus sphaericus*.

## Supporting Information

S1 Table Investigated phytoplankton groups biomasses (g WW m^-3^) in Lake Võrtsjärv during February 2010 to February 2011. Cyano—cyanobacteria; Bac—diatoms; Chloro—chlorophytes (green algae); Crypto—cryptophytes; Col cy—colonial cyanobacteria; Total BM—total phytoplankton biomass. ed—fractions of respective phytoplankton groups by sizes edible for zooplankton. All cryptophytes were of sizes edible for zooplankton. WW- wet weight.
(DOCX)

S2 Table Investigated zooplankton biomasses (g WW m^-3^) in Lake Võrtsjärv during February 2010 to February 2011. WW- wet weight.
(DOCX)

S3 Table The number of investigated zooplankton animals per sample and total amount of investigated phytoplankton pigments (tot PPig; ng ml^-1^) measured in zooplankton guts in Võrtsjärv. (DOCX)
