# Supplementary material for: Algal Diet of Small-Bodied Crustacean Zooplankton in a Cyanobacteria-Dominated Eutrophic Lake
Source: PLoS One. 2016 Apr 28;11(4):e0154526. doi: 10.1371/journal.pone.0154526 (PMC4849668; doi:10.1371/journal.pone.0154526)
Supplement: S1 Table — Cyano—cyanobacteria; Bac—diatoms; Chloro—chlorophytes (green algae); Crypto—cryptophytes; Col cy—colonial cyanobacteria; Total BM—total phytoplankton biomass. ed—fractions of respective phytoplankton groups by sizes edible for zooplankton. All cryptophytes were of sizes edible for zooplankton. WW- wet weight. (DOCX) [file pone.0154526.s001.docx]

**S1 Table. Biomasses of investigated phytoplankton groups (g WW m^-3^) in Lake Võrtsjärv during February 2010 to February 2011.**

| **Date** | **Cyano** | **Bac** | **Chloro** | **Crypto** | **Col Cy** | **Total BM** | **Bac ed** | **Chloro ed** | **Col Cy ed** |
| --- | --- | --- | --- | --- | --- | --- | --- | --- | --- |
| 16.02.2010 | 1.356 | 0 | 0.002 | 0.013 | 0 | 1.4 | 0 | 0 | 0 |
| 16.03.2010 | 0.275 | 0 | 0.0004 | 0.006 | 0.007 | 0.3 | 0 | 0 | 0.007 |
| 20.04.2010 | 0.190 | 1.922 | 0.088 | 0.087 | 0.026 | 2.3 | 0.173 | 0.085 | 0.008 |
| 25.05.2010 | 2.235 | 6.189 | 0.368 | 0.149 | 0.042 | 8.9 | 0.161 | 0.358 | 0.006 |
| 16.06.2010 | 11.955 | 9.434 | 0.568 | 0.082 | 0.860 | 22.9 | 1.122 | 0.564 | 0.582 |
| 20.07.2010 | 16.879 | 5.307 | 0.117 | 0.054 | 0.305 | 22.7 | 0.665 | 0.110 | 0.039 |
| 26.08.2010 | 23.490 | 3.353 | 0.266 | 0.113 | 0.481 | 27.7 | 0.240 | 0.239 | 0.268 |
| 21.09.2010 | 26.691 | 7.338 | 0.611 | 0.023 | 0.175 | 34.8 | 0.051 | 0.587 | 0.175 |
| 19.10.2010 | 34.185 | 3.714 | 0.863 | 0.286 | 0.349 | 39.4 | 0.239 | 0.648 | 0.136 |
| 17.11.2010 | 11.309 | 1.784 | 0.152 | 0.324 | 0.255 | 13.8 | 0.282 | 0.148 | 0.148 |
| 15.12.2010 | 3.443 | 0.202 | 0.024 | 0 | 0.010 | 3.7 | 0 | 0.024 | 0.010 |
| 18.01.2010 | 1.893 | 0.054 | 0.004 | 0.004 | 0.038 | 2.0 | 0 | 0.004 | 0.029 |
| 15.02.2010 | 0.061 | 0 | 0 | 0 | 0.029 | 0.1 | 0 | 0 | 0.029 |

Cyano – cyanobacteria; Bac – diatoms; Chloro – chlorophytes (green algae); Crypto – cryptophytes; Col cy – colonial cyanobacteria; Total BM – total phytoplankton biomass. ed – fractions of respective phytoplankton groups by sizes edible for zooplankton. All cryptophytes were of sizes edible for zooplankton. WW- wet weight.
