# Supplementary material for: Algal Diet of Small-Bodied Crustacean Zooplankton in a Cyanobacteria-Dominated Eutrophic Lake
Source: PLoS One. 2016 Apr 28;11(4):e0154526. doi: 10.1371/journal.pone.0154526 (PMC4849668; doi:10.1371/journal.pone.0154526)
Supplement: S2 Table — WW- wet weight. (DOCX) [file pone.0154526.s002.docx]

**S2 Table. Investigated zooplankton biomasses (g WW m^-3^) in Lake Võrtsjärv during February 2010 to February 2011.**

| **Date** | ***Thermocyclops oithonoides*** | ***Mesocyclops leuckarti*** | ***Cyclops kolensis*** | ***Thermoc.+ Mesoc.***  **copepodites** | **Nauplii** | ***Chydorus sphaericus*** | ***Bosmina* spp.** | ***Daphnia cucullata*** | **Other**  **Cladocerans** | **Rotifers** |
| --- | --- | --- | --- | --- | --- | --- | --- | --- | --- | --- |
| 16.02.2010 | 0 | 0 | 0.144 | 0 | 0.020 | 0 | 0 | 0 | 0 | 0.009 |
| 16.03.2010 | 0 | 0 | 1.082 | 0 | 0.002 | 0 | 0 | 0 | 0 | 0.009 |
| 20.04.2010 | 0.019 | 0.011 | 0.179 | 0.026 | 0.007 | 0 | 0 | 0 | 0 | 0.012 |
| 25.05.2010 | 0.022 | 0.009 | 0.292 | 0.056 | 0.087 | 0.012 | 0.038 | 0 | 0 | 0.223 |
| 16.06.2010 | 0.078 | 0.084 | 0.189 | 0.157 | 0.026 | 0.186 | 0.075 | 0.065 | 0.003 | 0.190 |
| 20.07.2010 | 0 | 0.029 | 0 | 0.090 | 0.087 | 0.022 | 0.034 | 0.042 | 0.105 | 0.083 |
| 26.08.2010 | 0.012 | 0.140 | 0 | 0.160 | 0.057 | 0.016 | 0 | 0 | 0 | 0.041 |
| 21.09.2010 | 0 | 0.278 | 0.137 | 0.270 | 0.021 | 0.019 | 0.021 | 0 | 0.070 | 0.189 |
| 19.10.2010 | 0 | 0.103 | 0.213 | 0.056 | 0.003 | 0.061 | 0.013 | 0 | 0.003 | 0.060 |
| 17.11.2010 | 0 | 0 | 0.050 | 0.016 | 0.007 | 0.052 | 0 | 0 | 0.003 | 0.007 |
| 15.12.2010 | 0 | 0.0009 | 0 | 0 | 0.0004 | 0 | 0 | 0 | 0.0007 | 0.008 |
| 18.01.2010 | 0 | 0 | 0.021 | 0 | 0.005 | 0.0009 | 0 | 0 | 0.0002 | 0.004 |
| 15.02.2010 | 0 | 0 | 0.006 | 0 | 0 | 0 | 0 | 0 | 0 | 0.007 |

WW- wet weight.
