# Supplementary material for: Algal Diet of Small-Bodied Crustacean Zooplankton in a Cyanobacteria-Dominated Eutrophic Lake
Source: PLoS One. 2016 Apr 28;11(4):e0154526. doi: 10.1371/journal.pone.0154526 (PMC4849668; doi:10.1371/journal.pone.0154526)
Supplement: S3 Table — (DOCX) [file pone.0154526.s003.docx]

**S3 Table. The number of investigated zooplankton animals per sample and total amount of investigated phytoplankton pigments (tot PPig; ng ml^-1^) measured in zooplankton guts in Võrtsjärv.**

| **Date** | **ZP species** | **Numer of animals** | **Tot PPig (ng ml^-1^)** |
| --- | --- | --- | --- |
| 16.02.2010 | *Cyclops* *kolensis* 1 | ca 280 | 127 |
| 16.02.2010 | *C. kolensis* 2 | ca 280 | 121 |
| 16.03.2010 | *C. kolensis* 1 | ca 280 | 122 |
| 16.03.2010 | *C. kolensis* 2 | ca 280 | 121 |
| 20.04.2010 | *C. kolensis* 1 | ca 285 | 77 |
| 20.04.2010 | *C. kolensis* 2 | ca 285 | 123 |
| 11.05.2010 | *C. kolensis* 1 | 270 | 435 |
| 11.05.2010 | *C. kolensis* 2 | 290 | 409 |
| 11.05.2010 | *Thermocyclops oithonoides* | 400 | 83 |
| 25.05.2010 | *Mesocyclops leuckarti* 1 | 300 | 98 |
| 25.05.2010 | *M. leuckarti* 2 | 410 | 140 |
| 16.06.2010 | *C. kolensis* | 110 | 117 |
| 16.06.2010 | *T. oithonoides* | 200 | 22 |
| 16.06.2010 | *M. leuckarti* | 300 | 98 |
| 16.06.2010 | *Bosmina* spp. | 130 | 11 |
| 16.06.2010 | *Daphnia cucullata* | ca 100 | 8 |
| 06.07.2010 | *T. oithonoides* | 200 | 20 |
| 06.07.2010 | *M. leuckarti* | ca 300 | 55 |
| 06.07.2010 | *Bosmina* spp. | 100 | 9 |
| 06.07.2010 | *D. cucullata* | 250 | 16 |
| 20.07.2010 | *T. oithonoides* | 250 | 16 |
| 20.07.2010 | *M. leuckarti* | 330 | 58 |
| 20.07.2010 | *D. cucullata* | 290 | 14 |
| 20.07.2010 | *Chydorus sphaericus* | ca 345 | 19 |
| 03.08.2010 | *M. leuckarti* 1 | 300 | 64 |
| 03.08.2010 | *M. leuckarti* 2 | 300 | 73 |
| 03.08.2010 | *C. sphaericus* | ca 250 | 7 |
| 26.08.2010 | *M. leuckarti* | ca 300 | 54 |
| 08.09.2010 | *M. leuckarti* | 300 | 122 |
| 21.09.2010 | *C. kolensis* | 157 | 64 |
| 21.09.2010 | *M. leuckarti* | 200 | 80 |
| 05.10.2010 | *M. leuckarti* 1 | 300 | 70 |
| 05.10.2010 | *M. leuckarti* 2 | 260 | 43 |
| 19.10.2010 | *C. kolensis* | 250 | 149 |
| 19.10.2010 | *M. leuckarti* | 197 | 8 |
| 17.11.2010 | *C. kolensis* | 230 | 122 |
| 17.11.2010 | *M. leuckarti* | 225 | 7 |
| 17.11.2010 | *C. sphaericus* | ca 300 | 5 |
| 18.01.2011 | *C. kolensis* | 140 | 35 |
